# Relationship between Countermovement Jump and Sprint Performance in Professional Football Players

**DOI:** 10.3390/jcm13154581

**Published:** 2024-08-05

**Authors:** Łukasz Oleksy, Anna Mika, Maciej Kuchciak, Artur Stolarczyk, Olga Adamska, Miłosz Szczudło, Renata Kielnar, Paweł Wolański, Jarosław Michał Deszczyński, Paweł Reichert

**Affiliations:** 1 Department of Physiotherapy, Faculty of Health Sciences, Jagiellonian University Medical College Kraków, 31-008 Krakow, Poland; loleksy@oleksy-fizjoterapia.pl; 2 Department of Orthopedics, Traumatology and Hand Surgery, Faculty of Medicine, Wroclaw Medical University, 50-556 Wroclaw, Poland; pawel.reichert@umw.edu.pl; 3 Oleksy Medical & Sport Sciences, 37-100 Łańcut, Poland; 4 Institute of Clinical Rehabilitation, University of Physical Education in Kraków, 31-571 Kraków, Poland; 5 Department of Physical Education, University of Rzeszów, 35-959 Rzeszów, Poland; mkuchciak@ur.edu.pl; 6 Department of Orthopaedics and Rehabilitation, Medical Faculty, Medical University of Warsaw, 02-091 Warsaw, Poland; artur.stolarczyk@wum.edu.pl (A.S.); jm.deszczynski@me.com (J.M.D.); 7 Department of Ophthalmology, Faculty of Medicine, Collegium Medicum Cardinal Stefan Wyszyński University in Warsaw, 01-815 Warsaw, Poland; o.adamska@uksw.edu.pl; 8 Centre of Sport and Recreation, University of Rzeszów, 35-959 Rzeszów, Poland; mszczudlo@ur.edu.pl; 9 Institute of Health Sciences, Medical College of Rzeszów University, 35-315 Rzeszów, Poland; kielnarrenata@o2.pl; 10 Department of Physiology, Gdansk University of Physical Education and Sport, 80-336 Gdańsk, Poland; pawel.wolanski@awf.gda.pl; 11 Football Club, Academy Lechia Gdańsk, 80-560 Gdańsk, Poland

**Keywords:** countermovement jump, sprint test, football players, performance, training, injury risk

## Abstract

**Objectives:** The aim of this study was to assess the relationship between the countermovement jump (CMJ) and sprint performance of professional football players, and to determine which strength and speed elements assessed by the CMJ translate into effective running. **Methods:** The research sample comprised 87 male professional football players (age 23.7 ± 4.20 years; body mass 82.33 ± 6.56 kg; body height 1.86 ± 0.05 m) who performed the CMJ on a dual-force platform, as well as the 30 m sprint test. The time and velocity of the run were recorded by photocells at 0, 5, 10, and 30 m of the distance. **Results:** No significant differences were noted in the time or velocity of the sprint over the initial 5 m between the groups of football players with a higher and lower braking rate of force development (RFD) in the CMJ (*p* > 0.05). However, at subsequent intervals (5–10 m and 10–30 m), players with a higher braking RFD achieved significantly better time and velocity than those with a lower RFD. Significant correlations in the group with a lower braking RFD between the CMJ and sprint variables occurred in the propulsion phase of the CMJ and most of them were in the first interval (0–5 m). In the group with a higher braking RFD, significant relationships were visible in both the propulsion (concentric) and braking (eccentric) phases of the CMJ, mainly during the second and third intervals of the sprint test. **Conclusions:** The noted observations may suggest that the relationship between strength and running performance is more complex than previously indicated, and that higher strength in the CMJ does not fully correlate with better sprinting. Therefore, it has been hypothesized that training aimed at generally increasing strength may not always be fully beneficial for running performance in football players and hence specific training guidelines are suggested for targeted strengthening of the required muscle performance characteristics. This may possibly contribute to reducing the unnecessary muscle overload during both training and matches, thereby preventing sports-related injuries.

## 1. Introduction

In elite football, it has been found that a sprint bout occurs approximately every 90 s, with each sprint lasting 2 to 4 s, corresponding to 0.5–3.0% of the effective playing time. Moreover, above 90% of sprint bouts during a game are shorter than 30 m, with 49% being less than 10 m [1]. Therefore, rapid acceleration over short distances is crucial for these athletes [2,3,4]. In research on the subject, it has been indicated that greater muscular strength enhances performance in fundamental sports skills such as jumping, sprinting, and change of direction tasks, all essential components of football [1,2].

In previous studies, the rate of force development (RFD) has been defined as the rate of the rise in force over time, which is crucial across various sporting events [2,5,6]. In some studies, it has been suggested that sprint performance may be limited by the ability to produce a high RFD over brief contacts rather than absolute force application [2,7]. It has been demonstrated that professional sprinters generate higher vertical forces in the initial stance phase, highlighting the importance of the RFD in terms of sprinting ability [2,8]. It is widely assumed that foot contact during sprint running shares physio-mechanical similarities with movements such as the countermovement jump (CMJ) [9]. The effectiveness of a football player on the pitch largely depends on the ability to develop maximum running speed during the first few seconds, and this requires specific muscle performance in generating high acceleration [10]. However, despite numerous studies in this area, the relationship between lower limb strength and running speed in football players still involves many unexplained issues.

Tillin et al. [11] have shown that elite rugby players with a high RFD demonstrated substantially faster sprint times compared to those with a lower RFD, underscoring the importance of explosive strength in athletic performance. In previous investigations among elite and sub-elite sprinters, as well as in runners and elite football players [1], significant relationships were demonstrated between vertical jumping ability and sprint performance [12,13]. It was observed that stronger athletes tend to achieve faster sprinting performance compared to weaker subjects [1,9,14,15], although no significant difference between strong and weak athletes was confirmed in other studies [16,17].

Katja et al. [18] noted that the maximal velocity measured during the 60 m sprint, as well as the velocities at different intervals (0 to 10, 10 to 20 and 20 to 30 m), was correlated with the CMJ. Other authors have shown that greater power and strength in jumps may have beneficial effects on sprint performance [9]. However, it is not known whether this relationship is consistent across all athletes. Interestingly, Wisloff et al. [1] have indicated substantial time differences between football players for the 30 m sprint test. In particular, even if two of the players had similarly timed performances regarding the overall test, significant differences were observed between how they ran the first and last parts of the test. Therefore, it can be hypothesized that the total time of the running test may probably not be the best indicator of running performance among football players. The specificity of running during a match is based on short sprints and sudden accelerations; therefore, the running performance of a given player may be better demonstrated by the ability to accelerate in the first few seconds of the sprint. Thus, in the present research, the relationship between the force indicators from the CMJ and the running parameters in three intervals of the 30 m running test was analyzed.

Optimal adjustment of training methods to the specific demands of a given sport is a key element in both athletic performance and the prevention of sports-related injuries [19,20]. In prior research, it has been shown that factors such as insufficient or excessive muscle strength, or improper contraction speed and type, can lead to impaired motor control during movement. This, in turn, can cause overloads and injuries [21]. This study for the first time analyzed the relationship between CMJ and sprint performance when football players were divided into those with a higher and lower braking RFD measured during the CMJ, whereas other authors analyzed the general RFD without distinguishing its specific components. We have hypothesized that this approach may allow for a much deeper analysis of the sprint and jump performance and an evaluation of the nature of this relationship, which may imply further modifications to the training of football players. It may also be hypothesized that determining the detailed relationship between the CMJ variables and sprinting performance may be useful for modifying the training routines of football players in order to specifically strengthen those features allowing for more effective sprinting. Therefore, the aim of this study was to evaluate the relationship between the results obtained by football players in the CMJ jump and the performed sprint, and to determine which strength and speed elements assessed by the CMJ translate into effective running.

## 2. Materials and Methods

### 2.1. Participants

This study included 87 male football players aged 18–31 years, all belonging to first league professional teams (age 23.7 ± 4.20 years; body mass 82.33 ± 6.56 kg; body height 1.86 ± 0.05 m; training experience 10 ± 3 years). The inclusion criteria were as follows: no history of musculoskeletal injuries or being cleared to play by a medical specialist in the case of sustaining injuries in the past, playing professional football for at least 5 years, and no comorbidities that may influence the study results. The evaluation was conducted after obtaining agreement from the coaching staff. The data collection was preceded by a detailed conversation with the coach, who, based on the inclusion criteria, excluded players with injuries. Qualification for the study was managed by the coaching staff. The study participants were informed in detail about the research protocol and provided their written informed consent to participate in the study. Approval from the Ethical Committee of Regional Medical Chamber in Kraków was obtained for this study (35/KBL/OIL/2024). All the procedures were performed in accordance with the 1964 Declaration of Helsinki and its later amendments.

### 2.2. Procedures

The football players were asked to abstain from unaccustomed strenuous exercise for at least 24 h prior to testing. The protocol consisted of a single testing session during which anthropometric and demographic data (height, mass, age) were measured. A five-minute warm-up that included general dynamic exercises involving the entire body, such as light running, bends, lunges, and elements of dynamic stretching, was performed. The players knew the test protocol very well as they were routinely tested. However, to ensure that they were familiar and comfortable with the test protocol, they were allowed to practice it. For each test, a total of two successful trials were recorded, and the mean was used for further analysis.

#### 2.2.1. Countermovement Jump (CMJ)

Following the warm-up, participants performed the CMJ on the dual-force Hawkin Dynamics platform, which measured three-dimensional kinetic data bilaterally at a sampling rate of 1000 Hz (Hawkin Dynamics, Westbrook, ME, USA). The subjects started from an upright position, performing a rapid downward movement followed by dynamic complete extension of the lower-limb joints. To avoid undesirable changes in jump coordination, the athletes freely determined the amplitude of the countermovement. During the CMJ, participants were required to keep their hands on their hips throughout the full range of the movement. Each trial began with the participants standing still, with each foot placed on a force platform. Upon initiating the countermovement, participants attempted to jump vertically as high as possible. They were instructed to lower themselves as quickly as possible, jump as high as possible, and return to a standing position after landing [22]. The subjects performed two attempts at the CMJ, with a 10 s rest interval between jumps.

The following variables were analyzed:

- Peak Propulsive Velocity (m/s);
- Peak Relative Propulsive Power (W/Kg);
- Peak Relative Propulsive Force (%);
- Relative Propulsive Net Impulse (Newton Second per Kilo (N.s/Kg);
- Peak Braking Velocity (m/s);
- Peak Relative Braking Power (W/Kg);
- Peak Relative Braking Force (%);
- Relative Braking Net Impulse (Newton Second per Kilo (N.s/Kg);
- Braking RFD (N/s);
- Time to Take-Off (s);
- Take-Off velocity (m/s);
- Impulse Ratio (Unitless; Propulsive Net Impulse/Braking Net Impulse);
- Jump Height (m).

#### 2.2.2. Sprint Test

The sprint test consisted of a maximal running effort over a distance of 30 m The time and velocity of the run were recorded using the VALD Performance photocell system (VALD Performance Pty Ltd., Brisbane, Australia) located at the start (0 m), 5th, 10th meter and at the end (30th meter) of the distance. The system measures running and reaction times to the nearest 0.001 s.

The time and velocity were measured for three intervals of the distance:

- Between 0 and 5 m;
- Between 5 and 10 m;
- Between 10 and 30 m.

Based on the analysis of the receiver operating characteristic curve (ROC), the subgroups were distinguished. The Youden Index was calculated from the ROC curve. The analyses were conducted based on the division into stronger and weaker groups (using the variable braking RFD) and into faster and slower groups (using the sprint variable velocity 0–5 m). The braking RFD from the CMJ represents the RFD from the eccentric phase, which is a crucial element in force generation during the stretch-shortening cycle. The second variable, the velocity 0–5 m, represents the speed in the first phase of the sprint.

The participants classification with ROC methods involved the following steps (this process involved the following steps separately for the variable braking RFD and for the velocity 0–5 m):

1. Determining the threshold value: To classify participants into two groups, a threshold value for the continuous variables (braking RFD or velocity 0–5) was identified. This value was selected based on the ROC curve analysis, which allowed for the assessment of the point at which this variable best differentiates the two classes.

2. Calculating the ROC curve parameters: For each possible threshold value, the sensitivity and specificity were calculated. The ROC curve was created by plotting the sensitivity against (1—specificity) for various threshold values.

3. Optimal threshold value: The point on the ROC curve closest to the upper left corner of the plot (the point with the highest sensitivity and specificity) was considered the optimal threshold value. This allowed for maximal discrimination between the participants.

4. Classifying participants: After determining the optimal threshold value, participants were classified as belonging to one of the two groups. It was found that for the velocity 0–5 m variable, the optimal threshold value (cutoff point) on the ROC curve equal to 4.81 m/s was the most sensitive and specific measure separating individuals with a higher braking RFD (stronger) from those with a lower braking RFD (weaker). Subsequently, the value of the braking RFD equal to 7077 N/s (determined from the ROC curve), which most sensitively and specifically separated faster individuals from slower ones, was identified.

In further analyses, the following grouping variables were used:

- Velocity 0–5 m (4.81 m/s): Group 1a—faster, Group 2a—slower;
- Braking RFD (7077 N/s): Group 1b—higher braking RFD, Group 2b—lower braking RFD.

### 2.3. Statistical Analysis

STATISTICA 13.0 software was used for the statistical analysis. Data normality was tested with the Shapiro–Wilk test. The *t*-test was used to determine the differences between groups. The effect size (ES) was calculated using Cohen’s d and interpreted as small (0.2–0.3), medium (0.5) or large (>0.8) [23]. The variability within each dataset was described using the arithmetic mean and standard deviation (SD), coefficient of variation (CV) and standard error of measurement (SEM). The bias between two means (higher braking RFD vs. lower braking RFD group) was examined using the Bland–Altman method [24]. Additionally, Pearson’s linear correlation coefficient (r) was calculated. The sensitivity, specificity and receiver operator characteristic (ROC) curve were calculated. ROC curves plot the true-positive rate (sensitivity) against the false-positive rate (1 minus the specificity) for the possible cut-off score [25]. Differences were considered statistically significant at the level of *p* < 0.05. Power analysis determined that at least 35 subjects were required to obtain a power of 0.8 at a two-sided level of 0.05 with an effect size of *d* = 0.8. This analysis was based on data derived from the previous literature [1,13,14,15]. To ensure the better validity of this study, more participants were included.

## 3. Results

### 3.1. Comparison of Participants’ Basic Information

No significant difference was found for any variable concerning the participants’ basic information (Table 1)

### 3.2. Comparison of Sprint Test Variables between Football Players with Higher and Lower Braking RFD

There were no significant differences in the time or velocity of the sprint over the initial 5 m interval between the group of football players with a higher and those with a lower braking RFD. However, in the second (5–10 m) and third intervals (10–30 m), players with a higher braking RFD achieved a significantly better time and velocity than the group with a lower braking RFD. This means that athletes generating a higher braking RFD run faster, but not at the very beginning of the sprint (during 0–5 m interval). The football players with a higher braking RFD were slower during the first 5 m (they accelerated more slowly). However, in the subsequent intervals (5–10 m and 10–30 m), they achieved significantly better results than the players with a lower braking RFD (Table 2).

**Figure 1:**
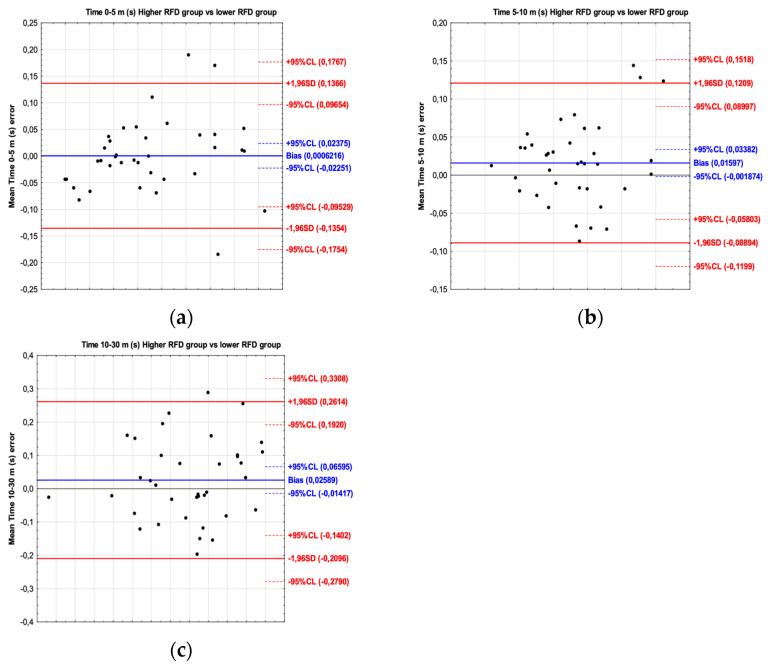
Bland–Altman plot showing the agreement between football players with a higher braking RFD and a lower braking RFD for the time 0–5 m (**a**), time 5–10 m (**b**) and time 10–30 m (**c**) sprint test intervals.

For the sprint time, the results of the Bland–Altman plot have shown bias between players with a higher and lower braking RFD. The mean difference for time 0–5 m was 0.0006 s (limit of agreement from −0.135 to 0.136); for time 5–10 m, the mean difference was 0.015 s (limit of agreement from −0.088 to 0.120); and for time 10–30 m, the mean difference was 0.025 s (limit of agreement from −0.209 to 0.261) (Figure 1).

**Figure 2:**
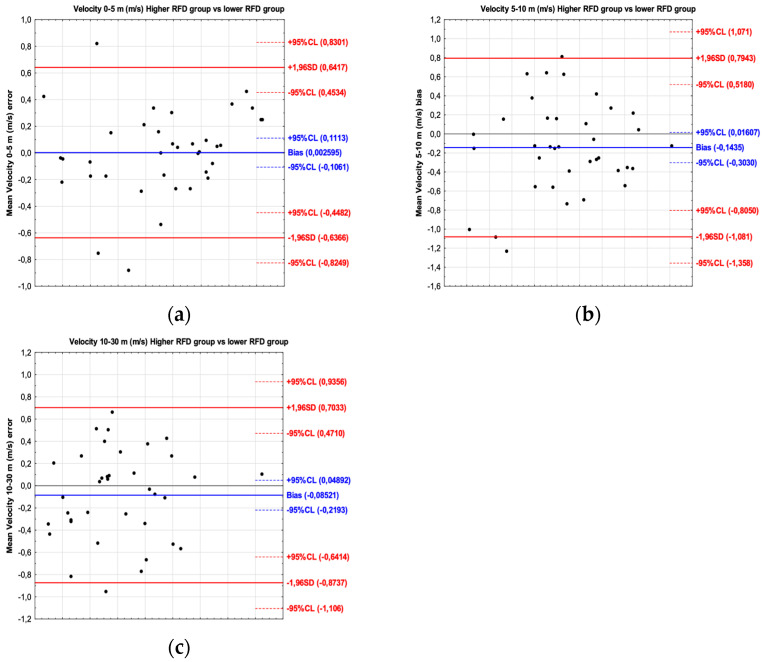
Bland–Altman plot showing the agreement between football players with a higher braking RFD and a lower braking RFD for the velocity 0–5 m (**a**), velocity 5–10 m (**b**) and velocity 10–30 m (**c**) sprint test intervals.

For the sprint velocity, the results of the Bland–Altman plot have shown bias between players with a higher and lower braking RFD. The mean difference for velocity 0–5 m was 0.002 m/s (limit of agreement from −0.636 to 0.641); for velocity 5–10 m, the mean difference was −0.143 m/s (limit of agreement from −1.081 to 0.794); and for velocity 10–30 m, the mean difference was −0.085 m/s (limit of agreement from −0.873 to 0.703) (Figure 2).

### 3.3. Comparison of CMJ Test between Faster and Slower Football Players

The football players who ran faster in the first 5 m interval (those with a lower braking RFD) performed worse in the CMJ. They had significantly lower propulsion and jump height results (the rest variables were also lower, but not significantly). On the other hand, the athletes who ran slower in the first 5 m interval (those with a higher braking RFD) had better CMJ results for all the variables, with significantly better results in the propulsion phase and jump height (Table 3).

### 3.4. Relationship between CMJ and Sprint Test Results

The correlations in the entire group, without dividing it into participants with a higher and lower braking RFD, were significant for the variables from the propulsion phase of the CMJ and for the jump height. Additionally, they were present in all three intervals of the sprint test (Table 4).

A similar pattern of significant relationships was observed in the group of football players with a lower braking RFD (who run faster in the initial meters), where most of the significant correlations between the CMJ and sprinting variables occurred in the propulsion phase of the CMJ. Significant correlations were present in all three intervals of the sprint test, but most of them occurred in the first interval (0–5 m) (Table 5).

In the group of football players with a higher braking RFD (who run slower in the initial meters), significant relationships were noted between the sprint and CMJ variables from both the propulsion (concentric) and braking (eccentric) phases of the CMJ. The significant correlations mainly occurred in the second and third intervals of the sprint test (Table 6).

## 4. Discussion

In the present research, it was demonstrated that among football players, better performance of the CMJ does not necessarily indicate superior sprinting ability. The current findings revealed that among players generating a higher braking RFD during the CMJ, slower velocity was observed in the initial meters of the sprint compared to athletes with a lower braking RFD in the CMJ. Furthermore, among players achieving higher speeds in the initial meters of the sprint (those with a lower braking RFD), the strongest correlations were observed between the variables from the propulsion phase of the CMJ and sprint velocity within the first interval (0–5 m), indicating that those footballers who better generate force in the concentric phase of the CMJ also accelerate better in the first few meters of the sprint. They probably utilize the force from the stretch-shortening cycle (SSC) more effectively and accelerate better initially, but they have worse performance within the later meters. This may suggest that high values of force in concentric contraction are crucial for effective running acceleration. However, in this study, individuals with higher braking RFD values, who accelerated more slowly, also demonstrated significant correlations between the running velocity and both the concentric and eccentric variables of the CMJ, but for the further meters of the running test. These observations may allow us to suggest that the relationship between strength and running performance is more complex than was indicated in previous studies, where greater strength in the CMJ was thought to strongly correlate with better sprinting.

It has been found that in professional football, more than 90% of sprint bouts during a match are shorter than 30 m, and almost 50% are less than 10 m [1]. It has been reported that within the initial two seconds, athletes may only reach approximately 70% of their maximal sprinting speed [2,26]. Therefore, the ability to accelerate rapidly and reach high sprinting speeds is critical during a football match. In several investigations, the relationship has been examined between sprinting ability and various strength measures [13,16,27,28,29]. In runners, a significant correlation (r = 0.6) has been reported between the maximum ground reaction force and the maximal sprinting velocity [30]. Also, in elite football players, a significant relationship has been observed between lower limb muscle strength and both acceleration and movement velocity [1,31]. Thus, it has been suggested that strength or maximal force production are integral components of the maximal sprinting velocity [1,8]. It has been indicated by some authors that stronger athletes demonstrate faster sprinting performances compared to those who are weaker [2,9,14,15], although in some studies, no differences have been found between strong and weak subjects [16,17].

In some studies, it has been suggested that sprint performance may be limited due to the ability to produce a high RFD over brief ground contacts rather than the ability to apply force [7]. Wisloff et al. [1] indicated that the maximal strength is strongly correlated with the RFD, so sprinting performance would also be related to an individual’s strength level. In previous research, it has also been shown that increases in strength coincide with improvements in short sprint performance [31,32,33,34]. Tillin et al. [11] have reported that elite rugby players who are strength-trained and exhibit a high RFD demonstrated notably faster 5 m sprint times and acceleration compared to athletes with a lower RFD. For the reason that acceleration depends mostly on the RFD, in many studies, a strong linear relationship has been indicated between the RFD and the sprint speed [1,9,14,15]. However, it the present study, these observations have not been fully confirmed. Nonetheless, it has been shown that better performance in the CMJ among football players did not translate into superior sprinting ability. This indicates that the relationship between strength and running speed is more complex than previously suggested, where greater strength in the CMJ was thought to always result in better sprinting. The current findings revealed that among the football players capable of generating a high braking RFD in the CMJ, lower running velocities were observed in the first interval of the sprint test compared to those with a lower braking RFD. Importantly, a higher braking RFD was the indicator of stretch-shortening efficacy, which is a crucial element of running performance itself but is not an indicator of acceleration during the first 5 m of the run. Football players with a higher braking RFD, indicating better dynamics and transition from the eccentric to concentric phases in the SSC, performed better in the later meters but not at the start of the sprint. Football players with a lower braking RFD, who were faster within the first 5 m, utilized the force from the SSC less effectively. As a result, they accelerated better initially but had worse performance within later meters. In other words, the better results achieved by football players with a lower RFD in the propulsion phase of the CMJ are likely due to the concentric contraction during the propulsion phase being assisted by the pre-stretching of elastic structures in the eccentric braking phase of the CMJ. Thus, the higher force values of the concentric contraction during propulsion are probably caused by the stretch-shortening cycle occurring here.

Wisloff et al. [1], in accordance with the observations in the present study, have also shown that a higher speed obtained in the running test does not mean that a given athlete is equally good at accelerating in the first few meters of the run. They have reported that among athletes who achieve similar total times for the 30 m sprint, significant differences existed in the times recorded for individual segments [1]. For some athletes, the best times were achieved in the initial meters, while for others, this occurred later in the sprint. They also suggested the implication of this finding to be that it is possible to tailor sprint training individually, focusing on split time recordings [1]. In the current research, it was also indicated that having a high braking RFD does not result in better acceleration during the initial meters of the run.

In several studies, the aim has been to identify potential predictors of sprinting performance through tests focused on strength–power parameters obtained from vertical and horizontal jumping and weightlifting assessments [13,28,35]. The underlying assumption was that the kinetic variables derived from these tests strongly correlate with the ability to rapidly produce force during sprinting [36]. One of the most commonly used types was the CMJ, which is used to measure the reactive strength under the stretch-shortening cycle [12,37,38]. It has been suggested that foot contact during running shows more physio-mechanical similarities to the CMJ [37]. The extra elastic energy stored in the elastic components of the muscle during the stretch-shortening processes helps to increase the running velocity in the concentric phase [12,37,38]. Significant relationships have been demonstrated between sprint parameters and the CMJ [1,9,12,13]. Kale et al. [12] reported that the CMJ was significantly correlated with performance in the 100 m sprint test (r = 0.46). Young et al. [30] stated that the CMJ was related to the maximum velocity due to its incorporation of the stretch-shortening cycle in its movement pattern. Berthoin et al. [39] found significant correlations between the maximum velocity and the CMJ (r = 0.56), and Faccioni et al. [40] also reported a significant correlation between the maximum velocity and the CMJ (r = 0.72) in elite sprinters. In the presented research, it has been indicated that the correlations in the entire group, without division into participants with a higher and lower braking RFD, were significant for the variables from the propulsion phase of the CMJ and for jump height, and they were further present during all three intervals of the sprint test. Similar relationships were observed in football players with a lower braking RFD (who run faster in the initial meters), where most of the correlations between the CMJ and the sprinting variables occurred in the CMJ propulsion phase, and in the majority of cases, during the first interval. Players with a lower braking RFD were faster at the beginning of the run and they accelerated better. On the other hand, football players with a higher braking RFD (who ran slower in the initial meters), demonstrated significant relationships between sprinting and CMJ variables for both the propulsion (concentric) and braking (eccentric) phases, and they occurred mainly during the second and third intervals of the sprint test. However, in the current study, the correlations were lower than those observed in other studies. The reason for this discrepancy may be data normalization. An important factor that may affect the obtained results is whether the CMJ force parameters were analyzed in relation to body mass or not. Non-normalized values may overestimate the results, which is why in this study, the relative values were analyzed. This issue was also underlined by other authors, who recommend that individual values of force production should be expressed relative to body mass to accommodate differences in anthropometric characteristics [13].

The results obtained in previous investigations demonstrated significant relationships between vertical jumping ability and sprint performance [1,9,12,13]. The conclusions from those studies supported the concept of maximizing lower-body strength to improve sprinting ability in athletes. Both the maximal strength and the RFD were indicated to be crucial for successful football performance due to the demands of the game. Moreover, a high level of strength and endurance capacity in football studies has allowed authors to suggest that both capacities are needed for optimal performance and should be intensively developed during daily training [1]. Nonetheless, in running, skillful generation of the RFD has been suggested to be more important than only possessing high strength [2]. Researchers have shown that during the first half of the support phase in running, athletes generate greater vertical and horizontal force, and a high RFD is crucial during this phase [2]. They suggest that a higher RFD translates onto a better maximal running speed [2,8,30]. In the current research, such a relationship has been confirmed, but not at the very beginning of the run. It becomes significant after approximately 5 m, when the runner has already attained a certain speed. Simply having a high braking RFD does not necessarily result in better acceleration during the initial meters of the run.

An essential aspect that should be underlined is that in the present study, the braking RFD measured during the CMJ test was analyzed, whereas other authors analyzed the general RFD without distinguishing its specific components. Therefore, considering the results of this study, the need for future research is suggested, in which the complex relationships between sprint and jump performance would be determined, but with consideration of the different ways to obtain and calculate the RFD. This could provide new perspectives on the nature of these relationships and imply further modifications to the training of these athletes.

This study also has some limitations that should be addressed. Only football players were evaluated; thus, more future research is required. It should include athletes from other disciplines. Also, a limitation of this study is the fact that only first league professional football players participated. Therefore, there is a need to check whether the same relationship exists among players at lower levels, female football players, and athletes in other team sports. Moreover, the study design was observational and the subjects were assessed only once; thus, longitudinal monitoring of jump and sprint performance would be of interest.

## 5. Conclusions

The observations in this study may allow us to suggest that the relationship between strength and running performance is more complex than previously indicated, and that higher strength in the CMJ does not fully correlate with better sprinting. Therefore, it has been hypothesized that training aimed at generally increasing strength may not always be fully beneficial for running performance in football players. Thus, it may be further suggested that those playing in positions where maximal acceleration within the first few meters is crucial should be focused on training that builds strength in conditions of concentric contraction, rather than solely emphasizing maximal overall strength gains. On the other hand, individuals playing in positions achieving maximal speed over longer distances should incorporate both eccentric and concentric elements into their strength training. This study, through a much broader analysis than previous works, allows us to indicate the specific training guidelines aimed at the targeted strengthening of those muscle performance characteristics that are required for individual players. This may contribute to reducing the unnecessary muscle overload during both training and matches, thereby preventing sports-related injuries.

## Figures and Tables

**Table 1:** Basic information differences of participants in the subgroups.

Outcome Measure	Group Velocity 0–5 m	Mean ± SD	*p*	Group Braking RFD	Mean ± SD	*p*
Body height (m)	1a	1.79 ± 0.04		1b	1.89 ± 0.03	
	2a	1.88 ± 0.05	0.87	2b	1.80 ± 0.06	0.97
Body mass (kg)	1a	80.21 ± 5.54		1b	83.30 ± 5.21	
	2a	82.83 ± 6.01	0.97	2b	79.45 ± 6.02	0.75
Age (years)	1a	22.3 ± 5.32		1b	23.1 ± 3.25	
	2a	24.7 ± 3.40	0.86	2b	24.3 ± 3.30	0.92

SD—standard deviation; *p*—*p* value; Group 1a = faster; Group 2a = slower; Group 1b = higher braking RFD; Group 2b = lower braking RFD.

**Table 2:** Comparison of the sprint test variables between football players with a higher and lower braking RFD.

Outcome Measure	Group	n	Mean ± SD	*p*	ES	CV	SEM
Time 0–5 m (s)	1b	50	1.02 ± 0.05	0.48	0.16	4.90	0.007
Time 0–5 m (s)	2b	37	1.01 ± 0.06			6.12	0.010
Time 5–10 m (s)	1b	50	0.72 ± 0.03	**0.01**	**0.60**	4.30	0.004
Time 5–10 m (s)	2b	37	0.74 ± 0.03			5.37	0.006
Time 10–30 m (s)	1b	50	2.41 ± 0.08	**0.04**	**0.44**	3.34	0.011
Time 10–30 m (s)	2b	37	2.45 ± 0.08			3.37	0.013
Velocity 0–5 m (m/s)	1b	50	4.91 ± 0.23	0.40	0.17	4.75	0.033
Velocity 0–5 m (m/s)	2b	37	4.96 ± 0.29			6.01	0.049
Velocity 5–10 m (m/s)	1b	50	6.93 ± 0.29	**0.01**	**0.53**	4.24	0.041
Velocity 5–10 m (m/s)	2b	37	6.76 ± 0.34			5.12	0.057
Velocity 10–30 m (m/s)	1b	50	8.28 ± 0.27	**0.04**	**0.43**	3.34	0.039
Velocity 10–30 m (m/s)	2b	37	8.16 ± 0.27			3.36	0.045

SD—standard deviation; *p*—*p* value; ES—effect size; CV—coefficient of variation; SEM—standard error of measurement; Group 1b = higher braking RFD; Group 2b = lower braking RFD.

**Table 3:** Comparison of the CMJ test variables between faster and slower football players.

Outcome Measure	Group	n	Mean ± SD	*p*	ES	CV(%)	SEM
Peak Propulsive Velocity (m/s)	1a	30	1.62 ± 0.14	**0.03**	**0.50**	8.78	0.02
Peak Propulsive Velocity (m/s)	2a	57	1.67 ± 0.09			5.47	0.01
Peak Relative Propulsive Power (W/Kg)	1a	30	51.8 ± 5.9	**0.02**	**0.50**	11.50	1.09
Peak Relative Propulsive Power (W/Kg)	2a	57	54.8 ± 5.5			10.05	0.72
Peak Relative Propulsive Force (%)	1a	30	259 ± 19	0.76	0.04	7.42	3.51
Peak Relative Propulsive Force (%)	2a	57	260 ± 25			9.64	3.33
Relative Propulsive Net Impulse (N.s/Kg)	1b	30	2.69 ± 0.21	**0.01**	**0.52**	7.85	0.03
Relative Propulsive Net Impulse (N.s/Kg)	2a	57	2.79 ± 0.17			6.06	0.02
Peak Braking Velocity (m/s)	1a	30	−1.28 ± 0.24	0.22	0.30	−18.88	0.04
Peak Braking Velocity (m/s)	2a	57	−1.36 ± 0.28			−21.00	0.03
Peak Relative Braking Power (W/Kg)	1a	30	−18.8 ± 5.3	0.22	0.28	−28.31	0.97
Peak Relative Braking Power (W/Kg)	2a	57	−20.7 ± 7.3			−35.45	0.97
Peak Relative Braking Force (%)	1a	30	247 ± 33	0.39	0.18	13.47	6.08
Peak Relative Braking Force (%)	2b	57	253 ± 33			13.21	4.44
Relative Braking Net Impulse (N.s/Kg)	1a	30	1.27 ± 0.24	0.21	0.30	18.87	0.04
Relative Braking Net Impulse (N.s/Kg)	2a	57	1.35 ± 0.28			21.04	0.03
Braking RFD (N/s)	1a	30	7340 ± 2731	0.43	0.18	37.21	498
Braking RFD (N/s)	2a	57	7862 ± 3058			38.89	405
Time to Take-Off (s)	1a	30	0.77 ± 0.08	0.25	0.27	11.29	0.01
Time to Take-Off (s)	2a	57	0.80 ± 0.11			14.06	0.01
Take-Off Velocity (m/s)	1a	30	2.67 ± 0.21	**0.01**	**0.55**	8.04	0.03
Take-Off Velocity (m/s)	2b	57	2.77 ± 0.17			6.10	0.02
Impulse Ratio (Propulsive Net Impulse/Braking Net Impulse)	1a	30	2.16 ± 0.41	0.94	0.02	19.03	0.07
Impulse Ratio (Propulsive Net Impulse/Braking Net Impulse)	2a	57	2.15 ± 0.50			23.48	0.06
Jump Height (m)	1a	30	0.36 ± 0.05	**0.01**	**0.53**	15.70	0.010
Jump Height (m)	2a	57	0.39 ± 0.04			12.09	0.006

SD—standard deviation; *p*—*p* value; ES—effect size; CV—coefficient of variation; SEM—standard error of measurement; Group 1a = faster; Group 2a = slower.

**Table 4:** Relationship between the CMJ and sprint test results in the entire group.

Outcome Measure	Time 0–5 m (s)	Time 5–10 m (s)	Time 10–30 m (s)	Velocity 0–5 m (m/s)	Velocity 5–10 m (m/s)	Velocity 10–30 m (m/s)
Peak Propulsive Velocity (m/s)	**−0.219**	−0.135	−0.126	**0.221**	0.143	0.128
Peak Relative Propulsive Power (W/Kg)	**−0.210**	**−0.282**	**−0.377**	**0.211**	**0.285**	**0.373**
Peak Relative Propulsive Force (%)	−0.146	−0.099	−0.123	0.144	0.093	0.120
Relative Propulsive Net Impulse (N.s/Kg)	**−0.253**	**−0.239**	**−0.317**	**0.254**	**0.248**	**0.316**
Peak Braking Velocity (m/s)	0.144	0.090	0.046	−0.137	−0.104	−0.044
Peak Relative Braking Power (W/Kg)	0.143	0.113	0.078	−0.136	−0.122	−0.075
Peak Relative Braking Force (%)	−0.107	−0.083	−0.046	0.103	0.086	0.047
Relative Braking Net Impulse (N.s/Kg)	−0.149	−0.092	−0.044	0.142	0.106	0.043
Braking RFD (N/s)	−0.099	−0.145	−0.072	0.091	0.144	0.073
Time to Take-Off (s)	−0.120	0.140	0.070	0.125	−0.145	−0.067
Take-Off Velocity (m/s)	**−0.263**	**−0.241**	**−0.309**	**0.265**	**0.250**	**0.308**
Impulse Ratio (Propulsive Net Impulse/Braking Net Impulse)	0.012	0.002	−0.102	−0.005	−0.014	0.103
Jump Height (m)	**−0.255**	**−0.239**	**−0.309**	**0.256**	**0.248**	**0.308**

**Table 5:** Relationship between the CMJ and sprint test results in the group of football players with a lower braking RFD.

Outcome Measure	Time 0–5 m (s)	Time 5–10 m (s)	Time 10–30 m (s)	Velocity 0–5 m (m/s)	Velocity 5–10 m (m/s)	Velocity 10–30 m (m/s)
Peak Propulsive Velocity (m/s)	**−0.350**	−0.064	0.061	**0.359**	0.081	−0.057
Peak Relative Propulsive Power (W/Kg)	**−0.281**	**−0.341**	**−0.420**	**0.280**	**0.356**	**0.421**
Peak Relative Propulsive Force (%)	**−0.212**	−0.119	−0.188	**0.349**	0.126	0.191
Relative Propulsive Net Impulse (N.s/Kg)	**−0.356**	**−0.301**	**−0.280**	**0.366**	**0.321**	**0.281**
Peak Braking Velocity (m/s)	0.208	−0.001	−0.134	−0.205	−0.021	0.138
Peak Relative Braking Power (W/Kg)	0.178	0.030	−0.134	−0.179	−0.050	0.140
Peak Relative Braking Force (%)	−0.214	0.069	0.212	0.231	−0.053	−0.209
Relative Braking Net Impulse (N.s/Kg)	−0.12	−0.002	0.132	0.209	0.025	−0.136
Braking RFD (N/s)	−0.132	0.111	0.328	0.146	−0.101	−0.327
Time to Take-Off (s)	−0.142	−0.100	0.006	0.124	0.090	−0.003
Take-Off Velocity (m/s)	**−0.367**	−0.302	−0.269	**0.365**	0.322	0.270
Impulse Ratio (Propulsive Net Impulse/Braking Net Impulse)	0.030	−0.146	−0.293	−0.029	0.134	0.299
Jump Height (m)	**−0.351**	**−0.309**	−0.279	**0.348**	**0.328**	0.279

**Table 6:** Relationship between the CMJ and sprint test results in the group of football players with a higher braking RFD.

Outcome Measure	Time 0–5 m (s)	Time 5–10 m (s)	Time 10–30 m (s)	Velocity 0–5 m (m/s)	Velocity 5–10 m (m/s)	Velocity 10–30 m (m/s)
Peak Propulsive Velocity (m/s)	−0.238	0.179	−0.113	0.249	−0.171	0.112
Peak Relative Propulsive Power (W/Kg)	−0.122	**−0.394**	**−0.346**	0.124	**0.392**	**0.340**
Peak Relative Propulsive Force (%)	−0.137	**0.296**	0.158	0.140	**−0.296**	−0.160
Relative Propulsive Net Impulse (N.s/Kg)	−0.135	−0.143	**−0.355**	0.137	0.149	0.353
Peak Braking Velocity (m/s)	0.061	−0.011	−0.155	−0.160	0.002	0.153
Peak Relative Braking Power (W/Kg)	−0.143	**−0.364**	**−0.333**	−0.144	**0.357**	**0.328**
Peak Relative Braking Force (%)	−0.276	**−0.383**	−0.121	0.276	**0.381**	0.122
Relative Braking Net Impulse (N.s/Kg)	−0.167	0.009	−0.052	0.166	−0.000	0.050
Braking RFD (N/s)	−0.187	0.113	0.066	0.190	−0.120	−0.066
Time to Take-Off (s)	−0.018	0.084	−0.213	0.030	−0.079	0.213
Take-Off Velocity (m/s)	−0.144	−0.146	**−0.352**	0.146	0.152	**0.349**
Impulse Ratio (Propulsive Net Impulse/Braking Net Impulse)	0.070	−0.050	−0.103	−0.068	0.043	0.102
Jump Height (m)	−0.146	−0.144	**−0.345**	0.149	0.150	**0.342**

## Data Availability

All the data generated or analyzed during this study are included in this published article.
